# Use of Tetra-ammonium Tetrakis(4-Sulphonato)Phenyl Porphyrin for *Pseudomonas* and *Bacillus* Cell Imaging

**DOI:** 10.1155/2010/697528

**Published:** 2010-07-26

**Authors:** V. Sujatha, Bharat Sridhar, Srinath Krishnamurthy, K. S. Vinod Kumar, K. Senthil Kumar, Pennathur Gautam

**Affiliations:** Center for Biotechnology, Anna University, Chennai 600025, India

## Abstract

The use of tetraammonium tetrakis(4-sulphonato)phenyl porphyrin (TPPS), a water-soluble anionic compound, as a stain to analyse bacterial cells using fluorescent microscopy was investigated. TPPS was effectively used to analyse two different bacteria: *Pseudomonas aeruginosa* and *Bacillus cereus*. The variation in brightness with varying concentrations of TPPS was studied. The patterns of variations for these bacteria were found to be the same, but with consistently higher brightness for *Bacillus cereus*.

## 1. Introduction

Fluorescent microscopy is a robust technique to study cells [1]. This technique requires tagging of cells with a fluorophore, which is a molecule that will fluoresce when light at appropriate wavelengths is incident on it. Fluorophores should have good photostability and high quantum yield, and when used to image live cells they should be noncytotoxic and stable in vivo [2, 3]. They should also have low susceptibility to photobleaching without losing their sensitivity to image the cells [4]. In order to overcome photon attenuation in living cells, fluorophores with long emission at the near-infrared (NIR) region are generally preferred [5].

At present, the most commonly used fluorophore to image bacterial cell is fluorescein, but owing to a variety of factors it is not a good choice [6]. Many other fluorophores are also in current use, and new fluorophores with high quantum yield and low cytotoxicity are also being continuously screened for imaging cells. 

**Figure 1:**
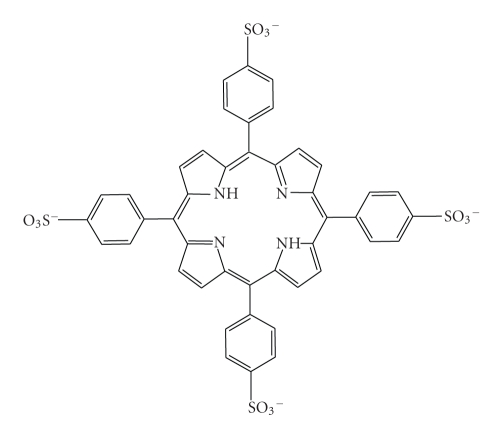
Tetrakis(4-sulphonato)phenyl porphyrin.

Porphyrin and its derivatives are nontoxic organic compounds whose fluorescent properties have been widely studied. Amphiphilic derivatives of porphyrin have been studied for second-harmonic generation-imaging [7]. We have investigated the use of tetra ammonium tetrakis (4-sulphonato)phenyl porphyrin (TPPS) (Figure 1), a water-soluble anionic compound, with multiple excitation bands and wide excitation range, as a fluorescent dye for imaging bacterial cells. 

In earlier studies, TPPS has been used as a photosensitising agent for photo-dynamic therapy. It was found to be moderately effective against Gram-positive bacteria but had no cytotoxic effect on Gram-negative bacteria, even after prolonged exposure to light at high frequency. Studies with tetra-cationic phthalocyanine as photosensitizer have also shown that the entry of photosensitizer into the cytoplasm increases brightness of cells, which was observed in images obtained from fluorescence microscope. The uptake of photosensitizer was further found to be an important step in affecting cells' survival rate [8]. These indicate that uptake of TPPS into the cytoplasm by Gram-positive bacteria is higher than that by Gram-negative bacteria. 

We imaged Gram-negative bacteria, *Pseudomonas aeruginosa,* and Gram-positive bacteria, *Bacillus cereus, *on a fluorescent microscope using TPPS as fluorophore and then analysed the brightness data obtained from those images.

## 2. Experimental Section

All the chemicals were procured from SRL chemicals (Mumbai, India.) 

### 2.1. Synthesis of TPPS

Tetraphenyl porphyrin was synthesized using the protocol described by Gonsalves et al. [9] followed by sulphonation using chlorosulphonic acid as described by Song et al. [10]. Purity of the compounds synthesised is verified using Thin-Layer Chromatography, NMR, UV visible spectroscopy, and mass spectrometry [11].

### 2.2. Cultures Used for Imaging

*Pseudomonas aeruginosa *(Gram-negative bacteria) (Genbank no. EU732606) and *Bacillus cereus *(Gram-positive bacteria) used were isolated and cultured in our laboratory.

Cultures of *Pseudomonas aeruginosa* were grown overnight and inoculated in Luria-Bertani medium, incubated for 14 hours. 250 *μ*L of this culture was mixed with various concentrations of TPPS and incubated in dark for 5 minutes. The cells were then centrifuged for 5 minutes at 5000 rpm. The cell pellet was washed and then solubilised in PBS buffer before being imaged on an Olympus X171 Total Internal Reflection Fluorescence microscope in fluorescence mode. The sample was excited using light from a mercury-vapour lamp, which was passed through an Olympus U-MWIBA3 filter (excitation filter 460 nm–495 nm and emission filter 510 nm–550 nm). Fluorescence was detected using an Andor iXON EMCCD camera.

*Bacillus cereus* was inoculated in nutrient broth and grown for 14 hours. After that, a similar procedure as used for *Pseudomonas aeruginosa* was followed and images were obtained. 

### 2.3. Image Analysis

Images collected from the Andor iXON EMCCD camera were processed using Andor-iQ software version 1.8. All images were taken at an exposure time of 117.5 ms and Real EM gain of 44. The image was smoothened and a threshold operation was performed. Following Image J, another image processing software was used to measure the brightness of cells in different parts of the image [12]. A mean value was then calculated. The default mode was used for making measurements. The weighted formula used to calculate brightness was

(1) V=0.299R+0.587G+0.114B,

where *R*, *G*, and *B* are the Red, Green, and Blue pixels, respectively.

A plot was then constructed to analyse the variation of brightness with changes in concentration of TPPS.

## 3. Results and Discussions

**Figure 2:**
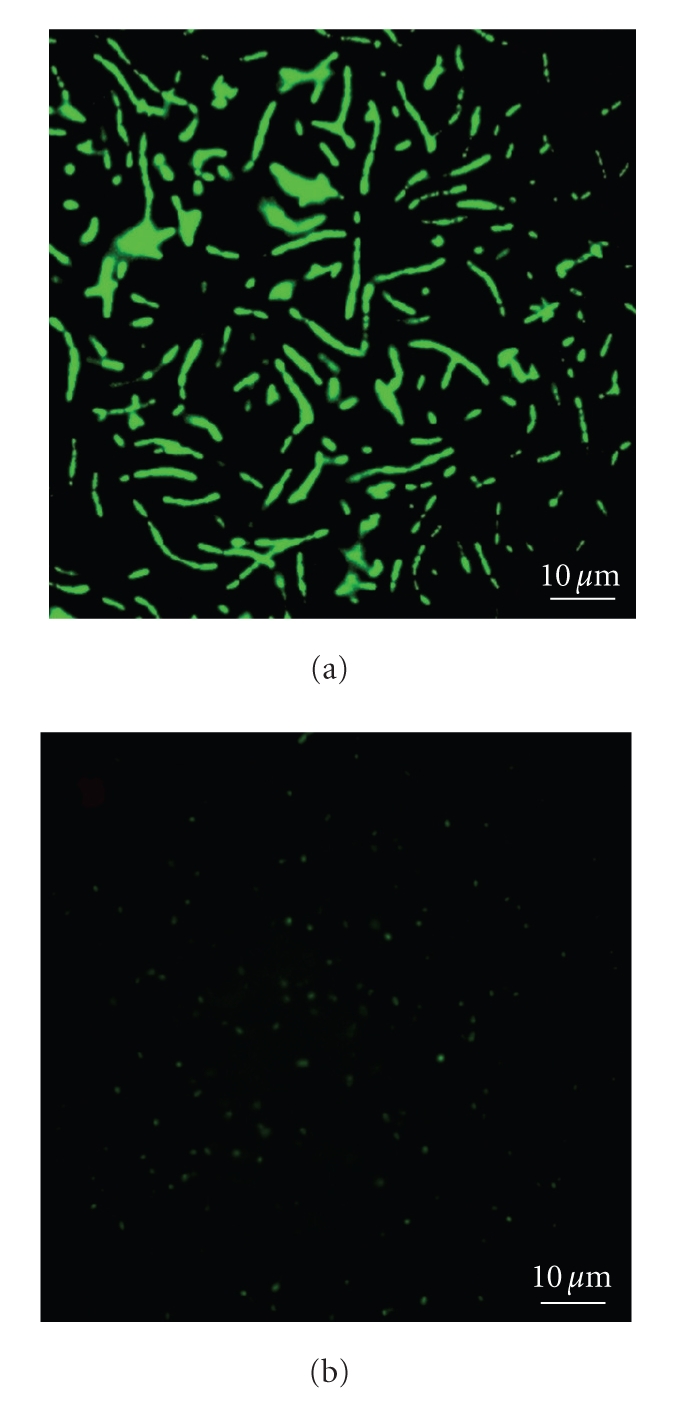
Fluorescent microscopy images of *Bacillus cereus* (a) and *Pseudomonas aeruginosa *(b).

The cells with TPPS fluoresced, when visualised under fluorescent microscope (Figure 2) with excitation at 488 nm wavelength, while control cells to which TPPS was not added did not show any fluorescence. 

Cell viability, for the *Pseudomonas aeruginosa* cells, as indicated by their motility, was not affected by the uptake of TPPS (Supplementary Material available online at doi:10.1155/2010/697528). 

**Figure 3:**
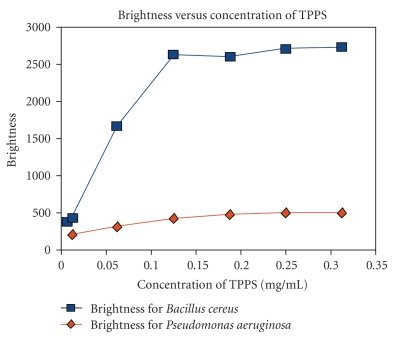
Variation of Brightness with concentration of TPPS for *Bacillus cereus *and *Pseudomonas aeruginosa *cells.

Image analysis revealed that the brightness increases with increasing concentrations of TPPS for both *Pseudomonas aeruginosa* and *Bacillus cereus* before achieving saturation. The concentration at which the brightness achieves saturation is about the same for both OF the bacteria (0.15–0.25 mg/mL), but the brightness is consistently higher for *Bacillus cereus* (Figure 3). This indicates that the uptake of TPPS by the Gram-positive bacteria is much higher than that of the Gram-negative bacteria. 

## 4. Conclusions

High quantum yield, high solubility in water, and its nontoxic nature are properties that confer considerable advantages to TPPS over other dyes in current use. The technique developed has the added advantage in that it requires a very short preparatory time. A variation in brightness was observed for the two different bacteria at the same concentrations of TPPS.

In conclusion, TPPS can be used as a stain for fluorescent imaging and analysis of various microorganisms and could be used for identifying *Pseudomonas aeruginosa* and *Bacillus cereus*. 

## Supplementary Material

The effect of TPPS on bacterial cells and its viability was analyzed. Over night grown culture of Pseudomonas aeruginosa was mixed with minimal amount of TPPS and was incubated for 12 hours. The viability of the organism does not seem to have affected by the uptake of TPPS. The video shows the mobility of Pseudomonas aeruginosa which fluoresce due to the uptake of TPPS.
